# Head, Face and Neck Cooling as Per-cooling (Cooling During Exercise) Modalities to Improve Exercise Performance in the Heat: A Narrative Review and Practical Applications

**DOI:** 10.1186/s40798-022-00411-4

**Published:** 2022-01-29

**Authors:** Yinhang Cao, Tze-Huan Lei, Faming Wang, Bin Yang, Toby Mündel

**Affiliations:** 1 School of Physical Education and Sport Training, Shanghai University of Sport, Shanghai, 200438 China; 2 College of Physical Education, Hubei Normal University, Huangshi, 435002 China; 3 School of Energy and Environment, Southeast University, Jiulong Lake Campus, Nanjing, 211189 China; 4 School of Energy and Safety Engineering, Tianjin Chengjian University, Tianjin, 300384 China; 5 School of Sport, Exercise and Nutrition, Massey University, Palmerston North, New Zealand

**Keywords:** Endurance performance, Heat stress, Thermoregulation, Pre-cooling, Per-cooling, Cooling intensity

## Abstract

It is well known that uncompensable heat stress greatly impairs endurance and team sport-related performance because an increase in the core temperature directly induces a greater magnitude of the central fatigue in the heat than in thermal neutral environments. Numerous studies have been conducted in an attempt to discover reliable cooling strategies for improving endurance performance and repeated sprint ability while exercising in the heat. Whole-body pre-cooling has been shown to improve endurance performance in both dry and humid heat. Despite this, the reduction in thermal perceptions associated with pre-cooling gradually narrows during intense exercise. Hence, effective per-cooling strategies to improve athletic performance in the heat are required. Unfortunately, due to practical issues, adopting pre-cooling approaches as a per-cooling (cooling during exercise) modality to improve athletic performance is impractical. Thus, we sought to examine the impact of head, neck and face cooling on athletic performance in heat. According to current evidence, cooling the head, neck and face reduced local skin temperature in the areas where cooling was applied, resulting in improved local perceptual sensations. In the heat, neck cooling during exercise improves athletic performance in both endurance and team sports athletes. Furthermore, from a practical standpoint, neck cooling is preferred over head, face and combined head/face and neck cooling for both endurance and team sport athletes in the heat. Nonetheless, for all athletes who have access to water, face cooling is a recommended cooling strategy. There is a lack of research on the systematic selection of per-cooling modalities to improve athletic performance based on environmental conditions and the nature of sports. In addition, powerful but portable head, neck and face cooling systems are urgently needed to assist athletes in improving their performance in hot conditions.

## Key Points

- Cooling the neck during exercise improves endurance performance in endurance athletes in the heat. Neck cooling during exercise also improves repeated sprint performance for team sport athletes in the heat.
- Cooling the head, neck and face during exercise reduces local skin temperature and thus improves local perceptual responses.
- Face cooling improves athletic performance and perceptual responses for athletes in dry heat. Thus, for all athletes who have access to water, face cooling is a recommended cooling strategy.
- Neck per-cooling (cooling during exercise) is preferred over head, face and combined head/face and neck per-cooling for endurance athletes.

## Introduction

The current consensus [1, 2] clearly indicates that performing endurance exercise in uncompensable heat stress environments imposes a greater thermoregulatory strain than performing endurance exercise in a cool or thermoneutral environment. This increase in thermoregulatory strain is primarily caused by the inability to dissipate metabolic heat through both dry and evaporative heat transfer, resulting in a higher core temperature [2]. A higher core temperature increases both thermal perceptual and cardiovascular strain, resulting in voluntary power output reduction [3] or premature fatigue as measured by the time to exhaustion approach [4].

Whole-body pre-cooling with water immersion has been found to improve endurance performance in both dry and humid heat as measured by time to exhaustion [5, 6] or by using the self-selected pace [7, 8]. This improvement in endurance performance is primarily due to physiological alterations (i.e., lower resting core and skin temperatures) caused by pre-cooling [5] which reduces cardiovascular and perceptual strain when performing prolonged endurance exercise in the heat. These improvements in thermoregulatory function, combined with lower perceptual strain, greatly improve endurance performance in uncompensable heat stress [9]. Nevertheless, the major drawbacks of whole-body pre-cooling are that it takes time and cannot be implemented during exercise. Furthermore, it is common that the desired physiological status (e.g., lower resting core temperature and thermal perceptions) induced by different whole-body pre-cooling regimes gradually narrows during exercise in uncompensable heat stress environments. Recently, previous studies have shown that when exercising in uncompensable heat stress (i.e., humid heat), per-cooling (cooling during exercise) is just as important as pre-cooling because it reduces thermal perceptions during exercise [10, 11]. Therefore, it is now recommended that pre-cooling is used in conjunction with a per-cooling (cooling during exercise) regime when exercising in the heat.

To date, per-cooling (cooling during exercise) has been used to reduce perceptual strain during endurance or team sport events in both dry and humid heat. Per-cooling (cooling during exercise) strategies have frequently been used to cool the upper body using a cooling vest or in local body regions such as the torso, head, face and neck. Although cooling the upper body with a cooling vest improves performance in laboratory settings, this method of per-cooling is impractical in the real-world situations due to the excessive weight of the vest (1 kg or higher). In contrast, cooling the head and neck regions is an effective way to mitigate perceptual strain and to improve physical performance in the heat and is relatively easy to wear with limited weight bearing when compared to cooling vests [12–16]. These two cooling interventions are based on the fact that the human body’s head/face and neck regions have greater alliesthesial sensitivity than the rest of the body, which can result in an immediate reduction in whole-body thermal discomfort when a cold stimulus is applied to mildly heat-stressed individuals [17]. This reduction in thermal perceptions improves both endurance and repeated sprint ability in the heat without altering the thermoregulatory response [18]. Furthermore, the cooling requirements of these regions are relatively small while providing ergogenic benefits similar to those of using whole-body pre-cooling via water immersion. As a result, many practitioners around the world are grappling with how to successfully incorporate per-cooling on top of whole-body pre-cooling.

Although previous studies [10, 13–15, 18] have clearly demonstrated that both head/face and neck cooling interventions can effectively improve athletic performance in the heat, those studies do not specifically address whether those interventions can be applied to both team sports and endurance athletes. Furthermore, there have been few studies that describe the physiological and performance differences between such cooling interventions. Finding a specific per-cooling strategy for different athletes is critical for recent and upcoming sporting events, e.g., the 2021 Tokyo Olympic Games and the 2022 soccer World Cup in Qatar, because it can improve physical performance while reducing thermoregulatory strain in uncompensable heat stress. The purpose of this narrative review is to specifically discuss the best per-cooling (cooling during exercise) strategies for various sports, such as endurance and team sport events. Furthermore, this narrative review provides an overview of the thermoregulatory and perceptual responses of each cooling intervention, which may provide valuable recommendations to the public regarding which cooling intervention is preferred when performing endurance exercise or team sports events during the summer. Finally, we highlight unexplored issues that could spur more mechanistic research in this field.

## Literature Search Methods and Considerations

Literature searches were conducted in the PubMed, MEDLINE, Scopus, Google Scholar and ProQuest databases until September 2021. Keywords used in the search included neck cooling, carotid cooling, head cooling, face cooling, exercise, per-cooling, pre-cooling, post-cooling, personal cooling, endurance performance, repeated sprint and time trial. Studies were included if the following criteria were met:

- Participants were described as “healthy” or “active,” and there were no known diseases that could impair exercise performance or thermoregulation;
- Studies were conducted at a temperature of ≥ 20 °C;
- Studies examined at least one type of cooling strategy applied to the head, face or neck or any possible combination of these three body regions;
- Studies reported either physiological or perceptual responses, or both;
- Studies were published in English in peer-reviewed journals, conference proceedings or published theses.

We excluded studies that used head, face and/or neck cooling modalities during occupational activities such as firefighting, farming or construction work. Any study that did not report the test condition was also disqualified. We also excluded two older studies from the 1970s and 1980s because we did not have access to the full texts and were unable to contact the authors to obtain the full texts.

Our search yielded 32 results for “neck cooling” AND “exercise” AND “healthy,” 24 results for “face cooling” AND “exercise” AND “healthy,” 22 results for “head cooling” AND “exercise” AND “healthy,” 15 results for “head and face cooling” AND “exercise” AND “healthy,” 9 results for “head and neck cooling” AND “exercise” AND “healthy” and 7 results for “face and neck cooling” AND “exercise.” Sixty-four of the 109 references did not address the areas of interest. Five relevant conference proceeding articles/extended abstracts and 1 relevant published thesis were discovered in the Google Scholar and ProQuest databases. Ultimately, 51 studies were included in this narrative review.

**Fig. 1:**
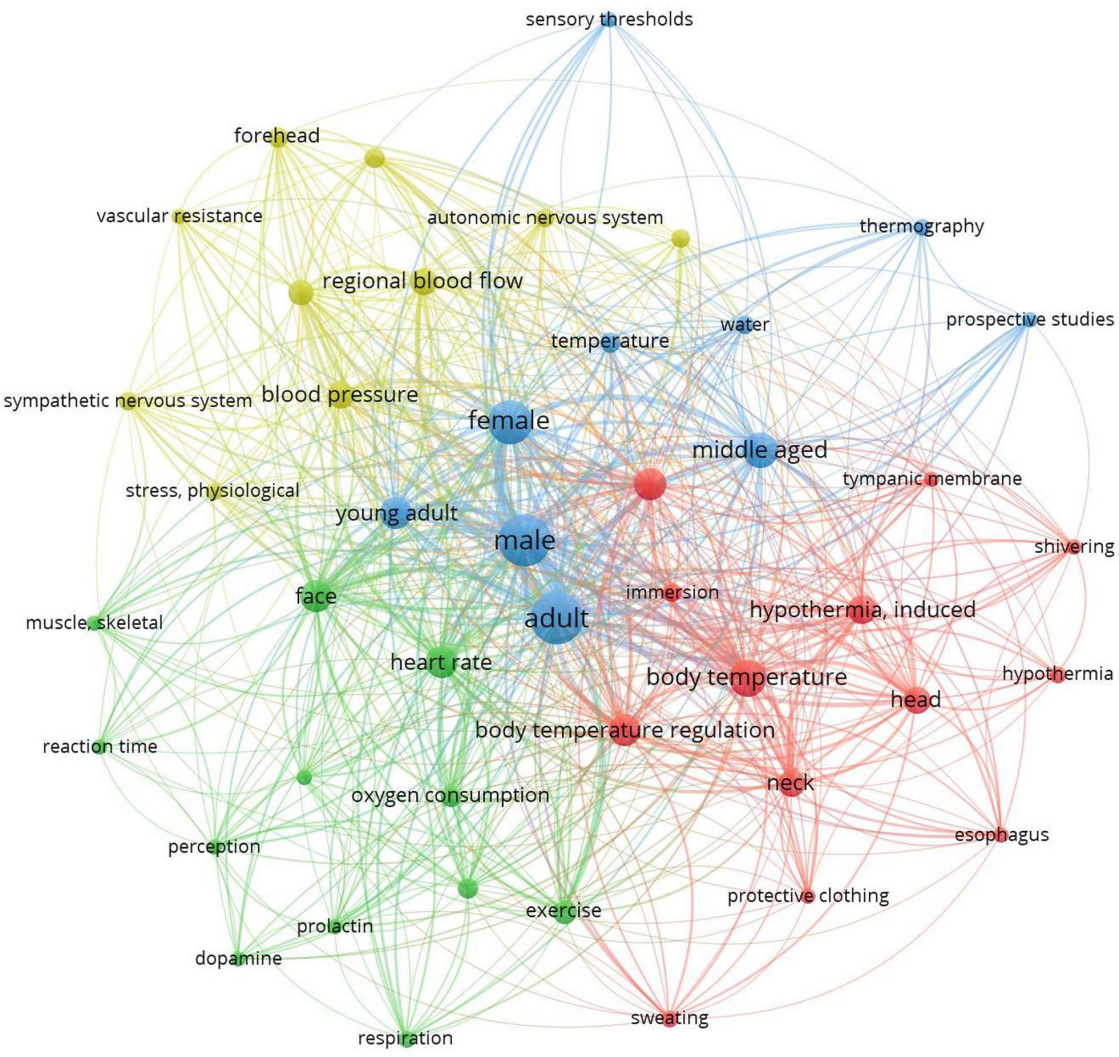
Co-occurrence map of frequently used keywords from the 51 published references

Figure 1 depicts the co-occurrence map of the most frequently used keywords in the abstracts of the 51 studies using VOSviewer (software version 1.6.17, Leiden University, The Netherlands). The co-occurrence map depicts the extent to which keywords occur concurrently in the reviewed keyword volume. The connection between any two concurrent keywords is represented by the network, while the frequency of a specific keyword is represented by its circle size. The dense network shows that keywords such as “male,” “female,” “face,” “head,” “neck,” “body temperature,” “skin temperature” and “heart rate” were frequently used and correlated with almost every other keyword. Furthermore, “adult” subjects were used as research subjects in the vast majority of documented studies. Furthermore, physiological parameters have been extensively studied, but there is very little information on perceptual responses such as thermal sensation (TS), thermal comfort/discomfort, thermal acceptance and ratings of perceived exertion. Future per-cooling studies should include females and young adults or even aged athletes as research participants. In addition to physiological parameters, perceptual responses while using per-cooling devices should be systematically examined and reported.

## Neck Cooling and Exercise Performance

Table 1 summarizes the published research on neck cooling and its performance on athletes while exercising in heat [13–15, 18–36]. Cooling collars, wet towels and ice bags were the most commonly used neck cooling approaches in the 23 documented studies. First, although six out of 23 studies did not report local neck temperature, the use of neck cooling could greatly reduce local skin temperature at the neck, and thus local thermal sensation at the neck was also significantly improved. A neck cooling collar with a − 80 ℃ cooling refrigerant has been found to improve both endurance and team sport performance in heat [13–15, 18, 19, 28–30]. This is demonstrated by the fact that wearing a cold neck collar improves time to exhaustion, self-paced performance and repeated sprint ability [13–15, 18, 19] when compared to no-cooling trials. This increase in physical performance with neck cooling may be due to the neck region’s high alliesthesial sensitivity and close proximity to the thermoregulatory center, namely, the insular cortex, which means that any cold stimulus can directly result in an immediate change in local thermal sensation (TS) and thus the rate of perceived exertion [17]. As a result, the self-selected pace or time to reach volitional exhaustion increases. The improved endurance performance with a neck cooling collar is primarily perceptually mediated, and its *T*_core_ response differs between time to exhaustion and the self-selected pace approach.

**Table 1:** Effect of neck cooling on thermoregulatory responses and physical performance in the heat

Study	Subjects	Ambient conditions	Cooling interventions	Exercise protocol	*T*_neck_	Perceptual outcomes	Thermoregulatory outcomes	Performance outcomes
Tyler et al. [13]	8 males	30 °C, 50% RH	Cooling collar (120 g gel, frozen at − 80 °C for 24–28 h [left in air 5 min before application])	15-min TT running	↓	↓ TS, → RPE	→ *T*_core_, → HR & SR, ↓ Wc	→ Distance (3180 vs. 3239 m)
Tyler et al. [13]	9 males	30 °C, 50% RH	Cooling collar (120 g gel, frozen at − 80 °C for 24–28 h [left in air 5 min before application])	Running 75 min at 60% VO_2max_ & 15-min TT running	↓	↓ TS, → RPE	→ *T*_core_, → HR & Bla & Gluc & Cortisol & Dopamine & Serotonin & S100β & PRL & PV & Wc	↑ Distance by 146 m (5%)
Tyler and Sunderland [14]	8 males	32.2 ± 0.2 °C, 53 ± 2% RH	Cooling collar (120 g gel, placed at − 80 °C for 24–28 h)	Running TTE at 70% VO_2max_	↓	↓ TS, → RPE	↑ *T*_core_, ↑ HR → SR & Wc & WL	↑ Time by 5 min (14%)
Tyler and Sunderland [15]	7 males	30.4 ± 0.1 °C, 53 ± 2% RH	Cooling collar (no change, or change every 30 min) (120 g gel, frozen at − 80 °C for 24–28 h [left in air 10 min before application])	Running 75 min at 60% VO_2max_ & 15-min TT running	↓	↓ TS, → RPE	→ *T*_core_ (a & b), → HR & Bla & Gluc & Wc & SR (a & b)	(a) ↑ Distance by 182 m (7%) (b) ↑ Distance by 179 m (7%)
Sunderland et al. [18]	7 males	33 ± 0.2 °C, 53 ± 2% RH	Cooling collar (120 g gel refrigerant frozen at − 80 °C for 24 h)	Repeated sprint exercise(5 × 6 s) before & after two 45-min bouts of football-specific intermittent treadmill protocol	↓	↓ TS & RPE	→ *T*_core_ & Tsk, → HR & Bla & Gluc & PRL & Cortisol & PV & Wc & WL	↑ Work load by 39 W (6%)
Minniti et al. [19]	8 males	30.5 ± 0.1 °C, 53 ± 2% RH	Cold collar (120 g gel, frozen at − 80 °C for 24–28 h)	Running 75 min at 60% VO_2max_ & 15-min TT running	NA	↓ TS & RPE	→ *T*_core_, → HR & VO_2_ & Gluc & Cortisol	↑ Distance by 153 m (5%)
Moss et al. [20]	9 males	40 °C, 50% RH	Cooling collar (155 g, 13.9 °C, replace every 15 min)	Cycling 45 min at 50% VO_2max_ followed by 15-min cycling TT	↓	→ TS & TD & RPE	→ *T*_core_ & Tsk, → HR	→ Distance
Bright et al. [21]	4 females & 8 males	34.4 ± 0.7 °C, 33 ± 1% RH	Cooling collar (frozen at − 1.3 °C replace every 20 min)	Cycling 90 min at RPE = 16	↓	↓ TS	→ *T*_core_ & Tsk, ↓ SR	→ Work load
Cuttell et al. [22]	8 males	35 ± 0.1 °C, 50.1 ± 0.7% RH	Cooling collar (155 g, frozen at − 24 °C for 45–60 min, 1.1% cooling area)	Cycling TTE at 60% PPO	↓	↓ TS, → RPE	→ *T*_core_ & Tsk, → RPE & HR & SR	→ Exercise time (30 vs. 28 min)
Galpin et al. [23]	13 males	25 ± 1 °C, 53 ± 1% RH	Wet ice bag (1 quart ice & 600 mL room-temperature water) was applied second times among three exercise bouts	Two, 5-min HEX bouts (20 s maximal cadence cycling, & 15 s passive rest) at 50% PP, & TTE at 30% PP	↓	↓ TS & RPE	→ HR & VO_2_	Exercise time (66 vs. 74 s)
Ando et al. [24]	8 males	35 °C, 70% RH	Wet towel (21 °C water & fanning back neck of with a small fan)	Ergometer cycling (30–32 W [first 5 min], increased at 20–21 W/min until HR = 160 bpm), cycling 10 min at 160 bpm	↓	→ RPE	↓ Tsk, → Bla & WL	→ Cognitive function during strenuous exercise
Lee et al. [25]	12 males	30.2 ± 0.3 °C, 71 ± 2% RH	Cooling collar (120 g gel refrigerant frozen at − 80 °C for > 24 h [left in ambient condition for 5 min before use])	Running TTE at 70% VO_2max_	↓	↓ TS, → RPE	→ *T*_core_ & Tsk, → HR & S100β & WL	→ Exercise time (71 vs. 67 min)
Desai and Bottoms [26]	8 male players	21.3 ± 3.4 °C, 44.5 ± 4% RH	Ice bag (310 g ice) was applied three times in each trial (1-min pre-exercise & after the 1st & 2nd bout)	Table-tennis-specific protocol (3 bouts, 660 s)	↓	→ TS & RPE	→ HR & VO_2_	↑ TPS (15%)
Zhang et al. [27]	6 males, 1 female	36 °C, 50% RH (running) & 20.7 °C, 45%RH (recovery & 6 × 15 m sprint & YYIR1)	Half-time cooling using a neck towel (placed in 5 °C ice water for 10 min)	45-min running & 15-min recovery	NA	↓ TS	→ *T*_core_ & HR, ↓ SR & dehydration	↑ YYIR1 distance
Tyler and Sunderland [28]	9 females	30.5 ± 0.1 °C, 53 ± 2% RH	Cold collar (120 g gel, frozen at − 80 °C for 24–28 h)	Running 75 min at 60% VO_2max_ & 15-min TT running	↓	↓ TS & RPE	→ *T*_*c*ore_, → HR & SR & Wc	↑ Distance by 146 m (5%)
Colvin and Lokody [29]	1 triathlon runner	41 °C, 50% RH	Cooling collar (454 g, 18 °C)	Running TTE	↓	NA	↓ *T*_core_	↑ Time by 17 min (51%)
Gabrys et al. [30]	11 males	40 °C, 80% RH	Water cooling collar (300 cm^2^, Tin = 14.8 °C, Tout = 18.4 °C, flow rate: 1.2 L/min)	Graded cycloergometer exercise at 50 W, increased by 50 W every 3 min (till 200 W)	NA	↓ TD	→ *T*_core_, → VO_2_, ↑ SV & CO	↑ Work load by 50 W (33%)
Chalmers et al. [31]	12 football players	35 °C, 55% RH, WBGT = 30 °C	Ice towel (2.5 kg ice) was applied 2.5 min at the 30-min mark of each half	Simulated football match (45 min + 15-min rest + 45 min)	NA	→ TS, ↓ RPE	↓ *T*_core_, → Tsk, ↓ HR	NA
Hamada et al. [32]	7 males	30 ± 1 °C, 40% RH	Ice pack cooling at bilateral carotid (500 g, change every 10 min; applied at 20th min)	Cycling 40 min at 60% VO_2max_	↓	↓ TS	→ *T*_core_ & Tsk, → SkBF, ↑ HR, ↓ SR	NA
Torii et al. [33]	7 males	30 °C, 40% RH	Ice pack cooling at bilateral carotid after 20-min exercise	Cycling 40 min at 60% PPO	↓	↓ TS	→ *T*_core_ & Tsk, → HR & SKBF, ↓ T_ty_, ↓ SR	NA
Bouskill and Parsons [34]	8 males	39.9 ± 0.2 °C, 27.1 ± 0.3% RH	Water cooling collar (Tin = 18.7 ± 0.2 °C, Tout = 19.4 ± 0.7 °C, flow rate: 0.8 L/h)	50-min exercise (step test to 25 cm height at 60 bpm)	↓	→ TS	↓ Tsk, ↑ HR, → SR & WL	NA
Gordon et al. [35]	10 male athletes	21 °C, 66.9% RH	Cooling collar (T unknown)	Running 45 min at RPE = 15	NA	→ RPE	↓ *T*_core_, ↓ SR, → HR	NA
Kielblock et al. [36]	5 males	40 °C, 70.5% RH	Instant cold pack (T unknown)	54 W external work until Δ*T*_core_ = + 2 °C	NA	NA	→ *T*_core_, → HR & BP	NA

→ no change, ↑ increase, ↓ decrease, *Bla* blood lactate concentration, *BP* blood pressure, *CO* cardiac output, *Gluc* glucose concentration, *HEX* high-intensity exercise, *HR* heart rate, *NA* not available, *PPO* peak power output, *PRL* blood prolactin concentration, *PV* plasma volume, *RH* relative humidity, *RPE* rating of perceived exertion, *SkBF* skin blood flow, *SR* sweat rate, *SV* stroke volume, *T*_*core*_ core temperature, *TD* thermal discomfort, *T*_*neck*_ neck skin temperature, *TPS* total performance score, *TS* thermal sensation, *Tsk* skin temperature, *T*_*ty*_ tympanic membrane temperature, *TT* time trial, *TTE* time to exhaustion, *VO*_*2*_ oxygen uptake, *VO*_*2max*_ maximal oxygen uptake, *Wc* water consumed, *WL* weight loss, *YYIR1* Yo–Yo intermittent recovery level 1 test

When assessing endurance performance using the time-to-exhaustion approach, the time to volitional exhaustion with the neck cooling collar was significantly longer than that in the control trial. This, however, corresponds to a higher core temperature at the time of exhaustion [14], leading to the conclusion that neck cooling may increase the risk of heat-related illness by masking the extent of thermal strain perceived. However, this statement is debatable because this approach does not allow us to thermoregulate behavior, which is an important mechanism for controlling body temperature during exercise. Furthermore, time to exhaustion has poor face validity and cannot represent the actual endurance event due to its open-ended design. Neck cooling has been shown in studies using self-selected pace to increase overall power output without affecting core temperature, heart rate and neuroendocrinological response [13, 15]. This suggests that by using our behavioral response, we can counteract the false signal caused by neck cooling and thus avoid an excessive rise in core temperature in the heat. Therefore, neck cooling is an effective cooling method for improving the endurance performance in the heat. However, it is worth noting that neck cooling may not be effective if the duration of exercise is less than 15 min, as Tyler et al. [13] demonstrated that neck cooling was ineffective in improving physical performance in a 15-min time trial.

It is also worth mentioning that some studies (13 out of 23, Table 1) [13, 20–25, 31–36] show that neck cooling does not provide any ergogenic benefit when performing prolonged exercise in the heat. The large disparity can be attributed to the cooling intensity not being strong enough to change the rating of perceived exertion (RPE), which is widely thought to be the most important perceptual marker for determining central fatigue in the heat because it is directly correlated with arousal level [68]. Specifically, the *α*/*β* wave ratio increased during hyperthermia, which has been linked to decrease arousal levels and contributes to the inability to maintain the desired power output during prolonged exercise in the heat.

## Head Cooling and Exercise Performance

A summary of 11 published studies on head cooling and its impact on sports and exercise performance is listed in Table 2 [16, 37–45]. Surprisingly, only four studies examined local head temperature and all discovered a pronounced temperature reduction at the head. More than half of the studies (6 out of 11 studies) did not investigate athletes’ perceptual responses during head cooling. Published studies [38–41] suggested that head cooling could potentially improve endurance performance in the heat [38–41], but this may not apply to team sport athletes because current studies (see Table 2) have yet to investigate whether head cooling can potentially improve repeated sprint ability for team sport athletes in any given thermal environment. Furthermore, whether head cooling can improve endurance performance in heat is still debatable, with the majority of studies revealing that head cooling has no ergogenic benefits in heat. There are currently very few studies on the effect of head cooling on the self-selected pace in the heat. Such disparities in previous studies are related to the intensity of the cooling, which is directly related to the cooling materials being used inside the cooling cap or the cooling helmet. It is possible that the majority of previous studies used a low cooling intensity for their study design, which was insufficient to alter the RPE response during exercise in the heat. Conversely, some studies (Table 2) have found that when the cooling intensity (5 ℃ of water) is able to attenuate the rise of RPE, physical performance improves [16, 40]. Therefore, it is believed that head cooling with a higher cooling intensity could potentially alter our arousal level, affecting our physical performance. This particular notion, however, necessitates further justification.

**Table 2:** Effect of head cooling on thermoregulatory responses and physical performance in the heat

Study	Subjects	Ambient conditions	Cooling interventions	Exercise protocol	*T*_head_	Perceptual outcomes	Thermoregulatory outcomes	Performance outcomes
Ansley et al. [16]	9 males	27–29 °C, 40–60% RH	Head cooling (3 fans placed at 50 cm from the face and head + a mist of water was sprayed over the head at 30 s intervals)	Cycling TTE at 75% VO_2max_	↓	↓ RPE	→ *T*_core_, ↓ Tsk, → HR & Bla & Gluc & VO_2_ & VE, ↓ PRL	↑ Time by 21 min (51%)
Levels et al. [37]	10 cyclists	30 °C, 50% RH	Head cooling (neoprene-covered silicone cooling cap connected to a cooling machine. *T*_coolant_ = – 9 to – 10 °C)	15 km TT cycling at 2 W/body mass	NA	→ TS & TD & RPE	→ *T*_core_ & Tsk, → HR	→ Exercise time
CoeIho et al. [38]	15 males	35 °C, 50% RH	Head cooling (cotton cap containing a cold mixture [− 20.2 ± 1.8 °C] of water and alcohol gel, change every 7 min) for 20 min prior to exercise	5 km TT running	↓	→ TS & RPE	↓ *T*_core_, → Tsk, → HR & SR	↑ Time by − 2 min (7%)
Walters et al. [39]	22 males	35 ± 1 °C, 15 ± 3% RH	Head cooling (cooling fluid through tubing and neoprene cap, *T*_water_ = 5–10 °C)	Cycling 40 min at 65% VO_2max_ & 7-min recovery, graded exercise test (1 W increased every 2.5 s until exhaustion, cooling removed)	NA	→ RPE	→ *T*_core_, → HR & Bla,	↑ Work load by 13 W (4%)
Minett et al. [40]	10 male athletes	33.0 ± 0.7 °C, 33.3 ± 3.9% RH	Head cooling (ice towel soaked in water [5 ± 0.5 °C] before being placed over the head)	2 × 35-min exercise spells separated by 15-min recovery	NA	↓ TS & RPE	→ pH & Gluc & HCO_3_	↑ Distance by 43 m (4%)
Hyde [41]	7 males & 7 females	38.5 ± 1.5 °C, 37.5 ± 7.6% RH	Head cooling (cooling cap connected to a cooling machine, T unknown)	Six bouts treadmill exercise (3–4.5 mph at 5% inclination)	NA	NA	→ *T*_core_, → HR	NA
Desruelle and Candas [42]	7 males	36 °C, 29% RH	Head cooling (10 °C air to the hood, 12 m/s)	Cycling 35 min at 90 W	↓	NA	→ *T*_core_, ↓ Tsk, → SR	NA
Watanuki[43]	6 females	25 °C, 56% RH	Head cooling (inlet *T*_water_ = 15 °C with a flow of 1.2 L/min)	Cycling 25 min at 25% VO_2max,_ & cycling 25 min at 50% VO_2max_	↓	NA	↓ HR & CO & VO_2_	NA
Katsuura et al. [44]	10 males	30 °C	Head cooling (thermoelectric cooled water circulating through tubing, *T*_water_ = 15 °C)	Cycling 45 min at 40% VO_2max_	NA	NA	↑ *T*_core_, ↓ SR, → SkBF	NA
Katsuura et al. [44]	10 males	40 °C	Head cooling (thermoelectric cooled water circulating through tubing, *T*_water_ = 15 °C)	Passive heating	NA	NA	↓ *T*_core_, ↓ HR & VO_2_ & CO & SkBF	NA
Greenleaf et al. [45]	4 males	40.1 °C, 40% RH	Head cooling (liquid cooling headgear, T unknown)	Cycling 60 min at 45% VO_2max_	NA	NA	→ *T*_core_, → HR & PP & Osmotic & ES, ↑ PV, ↓ SR	NA

→ no change, ↑ increase, ↓ decrease, *Bla* blood lactate concentration, *CO* cardiac output, *ES* electrolyte shift, *Gluc* glucose concentration, *HR* heart rate, *NA* not available, *PP* plasma protein, *PPO* peak power output, *PRL* blood prolactin concentration, *PSI* physiological strain index, *PV* plasma volume, *RH* relative humidity, *RPE* rating of perceived exertion, *SkBF* skin blood flow, *SR* sweat rate, *SV* stroke volume, *T*_*core*_ core temperature, *TD* thermal discomfort, *T*_*head*_ head skin temperature, *TS* thermal sensation, *Tsk* skin temperature, *TT* time trial, *TTE* time to exhaustion, *VE* minute ventilation, *VO*_*2*_ oxygen uptake, *VO*_*2max*_ maximal oxygen uptake, *Wc* water consumed

## Face Cooling and Exercise Performance

Table 3 shows the results of face cooling and its performance. Nine of the eleven included studies examined either the forehead or the face temperature. Six studies [12, 46, 49–51, 54] examined local perceptual sensations (thermal sensation and thermal discomfort). Face cooling, such as the effect of neck and head cooling on the neck and head regions, has the potential to greatly improve thermal sensation and alleviate thermal discomfort at the face. For physiological outcomes, although three studies [12, 46, 55] have suggested that face cooling appears to improve athletic performance in the heat (Table 3), the effect of face cooling on prolonged endurance performance needs further investigation. This is supported by the fact that face cooling only reduced RPE at the end of the stage [56]. Such small decrements of RPE may not be sufficiently large to elicit behavioral adaptation in highly trained subjects as their perceptual responses differ from those of the untrained population [57]. Stevens et al. [46] found that even in moderately trained subjects, face cooling improved the self-paced running speed for the first 2 km but was soon nullified thereafter. Nonetheless, intermittent face cooling significantly improved forehead temperatures, thermal sensation and muscle activation during the 5-km running time trial. Furthermore, no research has been conducted to determine whether face cooling can potentially increase the time to exhaustion or time trial performance in well-trained endurance athletes. Team sport athletes appear to reap significant ergogenic benefits from face cooling, as Miyazawa et al. [54] demonstrated that intermittent face cooling, similar to the team sport situation, is capable of lowering RPE during exercise in heat. Riera et al. [55] provided additional evidence that face cooling during swimming could increase the peak swimming velocity during 400-m swim events. However, it is worth noting that cooling the face with room temperature water (22 ℃) had little effect on athletic performance, whereas cooling the face with cold water (1.2 ℃) greatly improved the peak swimming velocity [55]. However, it remains unknown whether face cooling with water can provide ergogenic benefits to athletes in humid heat [58]. It is possible to conclude that face cooling improves athletic performance and perceptual responses for athletes in dry heat. Thus, for all athletes who have access to water, face cooling is a recommended cooling strategy. Nonetheless, to maximize face cooling benefits, the water temperature and the frequency of intermittent face cooling should be carefully chosen.

**Table 3:** Effect of face cooling on thermoregulatory responses and physical performance in the heat

Study	Subjects	Ambient conditions	Cooling interventions	Exercise protocol	*T*_local_	Perceptual outcomes	Thermoregulatory outcomes	Performance outcomes
Schlader et al. [12]	12 males	35 °C, 48% RH	Face cooling (20 °C air blown at 0.74 m/s)	Fixed RPE = 16	↓ *T*_face_	↓ TS & TD, → RPE	→ *T*_core_ & Tsk, → HR & SR	↑ Total work by 34 kJ (18%)
Stevens et al. [46]	9 male runners	32.5 ± 0.1 °C, 33.9 ± 5.8% RH	Intermittent facial water spray (*T*_water_ = 22 °C spraying once per km during the TT [15 sprays in total])	5 km TT running	↓ *T*_forehead_	↓ TS, → RPE	→ HR & Bla & VO_2_ & VE & RER & SR, ↑ iEMG	↑ Time by − 36 s (2.4%)
Schlader et al. [47]	9 males & 1 female	24 ± 1 °C, 35 ± 15% RH	Face cooling (placing ice water bag on forehead, eyes & cheeks)	Passive heating	↓ *T*_forehead_	NA	→ HR & CO & SkBF, ↓ BP	NA
Schlader et al. [48]	3 females (face-cooling) & 6 females (Sham trials)	24 ± 1 °C, 35 ± 15% RH	Face cooling (placing ice water bag on forehead, eyes & cheeks)	Supine position	↓ *T*_forehead_	NA	↑ BP & FVR, → HR & CO	NA
Mündel et al. [49]	10 males	33 °C, 27 ± 1% RH, fan speed: 0.5 m/s	Face cooling (spraying cold water mist [4 °C] to maintain *T*_face_ < 28 °C)	Cycling 40 min at 77–78% VO_2max_	↓ *T*_forehead_	→ TS, ↓ RPE	↓ *T*_core_, ↑ Tsk, ↓ HR & Dyspnea & PRL & Bla, → VO_2_ & WL & SR & Gluc & PV	NA
Mündel et al. [50]	12 males & 4 females	58 ± 1 °C, 13 ± 3% RH sauna	Face cooling (spraying ice-water mist [4 °C] for 10 s at 5-min intervals)	Passive heating	↓ *T*_forehead_	↓ TS	→ *T*_core_, ↓ Tsk, → HR, ↓ PRL	NA
Armada-da-Silva et al. [51]	10 males	35 ± 1 °C, 20% RH	Face cooling (cold water mist using electric fan)	Cycling 14 min at 63% MPO	↓ *T*_face_	→ TD, ↓ RPE	→ *T*_core_ & Tsk, → HR & Gluc & Hematocrit, ↓ RPE legs & RPL	NA
Williams and Kilgour [52]	5 males	Not mentioned	Face cooling ( cold wind [0 ± 2 °C] from air conditioning unit via insulated tube [0.2 m diameter])	Supine cycling 30 min at 35% VO_2max_ & 70% VO_2max_	↓ *T*_forehead_	NA	→ *T*_core_, ↓ Tsk, → SV & CO & VO_2_ & VE, ↓ HR, ↑ BP	NA
Kratzing & Cross [53]	6 males & 4 females	46 °C, low RH	Face cooling (cold air jet from tubing towards nose under a face mask)	Cycling 15 min at 25–32 km/h	NA	NA	→ HR & BP	NA
Miyazawa et al. [54]	10 males	35 °C, 50% RH	Face cooling (10 s at every 2 intervals or 4-min cooling during exercise (0 °C gel pack) or 10 s at every 2 min(20 °C gel pack)	65% peak power	↓ *T*_face_	↓ RPE	↓ *T*_core_, → Tsk, → HR & SR	NA
Riera et al. [55]	5 males & 3 females	29.6 ± 0.6C; 79 ± 10% RH	Face cooling (1.2 °C water or 22 °C water)	Five times 400 m swim	NA	→ TS, ↓ TD	→ *T*_core_,→ HR	↑swimming velocity

→ no change, ↑ increase, ↓ decrease, *Bla* blood lactate concentration, *BP* blood pressure, *CO* cardiac output, *FVR* forearm vascular resistance, *Gluc* glucose concentration, *HR* heart rate, *iEMG* integrated electromyography, *NA* not available, *PPO* peak power output, *PRL* blood prolactin concentration, *PV* plasma volume, *RER* respiratory exchange ratio, *RH* relative humidity, *RPE* rating of perceived exertion, *SkBF* skin blood flow, *SR* sweat rate, *SV* stroke volume, *T*_*core*_ core temperature, *T*_*local*_ local skin temperature, *TD* thermal discomfort, *T*_*face*_ face skin temperature, *T*_*forehead*_ forehead skin temperature, *TS* thermal sensation, *Tsk* skin temperature, *TT* time trial, *TTE* time to exhaustion, *VE* minute ventilation, *VO*_*2*_ oxygen uptake, *VO*_*2max*_ maximal oxygen uptake, *W*L weight loss

## Combined Head, Neck and/or Face Cooling and Exercise Performance

Table 4 shows the effects of combined face/head and neck cooling on sports and exercise performance [56, 59–64]. Clearly, combined head/face and neck cooling could largely improve thermal sensation and thermal discomfort at the cooling regions [56, 59–64]. The combined effect of either head and neck cooling or face and head cooling on exercise performance in the heat is unclear. Despite featuring a larger total cooling area in the combined cooling strategy when compared to the single cooling approach being applied to the face, neck or head, these aforementioned combined cooling methods may not provide any ergogenic benefits when compared to neck cooling alone [65]. The current literature has yet to address the combined effect of either head and neck cooling or face and head cooling on exercise performance in heat when compared to neck cooling alone for both endurance and team sport athletes. Furthermore, summarizing the current literature (Tables 1, 2, 3), it is suggested that the combined cooling may not actually provide any ergogenic benefit because the effect of both head and face cooling on both endurance and repeated sprint performance is equivocal. Finally, from a practical standpoint, combined cooling is thought to have less real-world application than neck cooling alone due to the difficulty of implementing this cooling intervention during exercise in the heat.

**Table 4:** Effect of combined neck, head and face cooling on thermoregulatory responses and physical performance in the heat

Study	Subjects	Ambient conditions	Cooling interventions	Exercise protocol	*T*_local_	Perceptual outcomes	Thermoregulatory outcomes	Performance outcomes
Simmons et al. [56]	6 males, 3 females	34 ± 1 °C, < 30% RH sauna temp = 68 ± 3 °C	Head & face cooling (Ice packs placed around whole head)	Two 12-min cycling tests at 70% VO_2max_ separated by a passive heating period in sauna	NA	↓ TS & RPE	↓ *T*_core_ & Tsk, → HR	→ Exercise time
Wiewelhove et al. [59]	8 male tennis players	31.8 ± 2.1 °C, 48.5 ± 9.6% RH	Neck & face cooling (ice-filled damp towel & electric fanning at 1 m distance)	45-min simulated tennis match	↓ *T*_neck_ & *T*_face_	↓ TS	↓ HR, ↑ RR	→ Exercise time
Palmer et al. [60]	14 male runners	33 °C, 55% RH	Head & neck cooling (water-perfused hood (1.1 L/min, 1 °C, 6.2 m PVC tubing)	Running 30 min at 60% VO_2max_ followed by 15-min running TT	NA	↓ TS & TC, → RPE	↓ *T*_core_, → HR	↑ Distance (3.3%)
Gordon et al. [61]	14 males	35 °C, 50% RH	Head & neck cooling (water-perfused hood & neck cooling, Tin = 3 °C)	Cycling 60 min at 50% VO_2max_	↓ *T*_neck_	↓ TS	→ *T*_core_, → MVC, ↑ PF, ↓ MVF & CAR & CF,	NA
Goosey-Tolfrey et al. [62]	8 wheelchair tennis players	30.4 ± 0.6 °C, 54 ± 3.8% RH	Head & neck cooling (water absorbing crystals, refreshed every 10 min, T unknown)	60-min intermittent sprint trials	NA	↓ TS & RPE	↓ Wc, → HR & Bla	NA
Simmons et al. [63]	6 males, 4 females	25 °C, 50% RH increased to 45 °C, 50%RH	Head & neck cooling (water conditioned balaclava, *T*_water_ = = 3 °C)	Passive heating	↓ *T*_neck_	↓ TD	↓ HR, ↑ SV, → CO	NA
Ronald et al. [64]	10 males, 10 females	30 ± 1 °C, 54 ± 5% RH	Head & neck cooling (evaporating ice water absorbed by material [poplin fabric cover & ties placed at forehead & neck bandanna tied around neck])	Cycling 30 min at 60% VO_2max_	↓ *T*_head_	→ RPE	→ *T*_core_ & Tsk, → HR & VO_2_ & SR & PV & BP	NA

→ no change, ↑ increase, ↓ decrease, *Bla* blood lactate concentration, *BP* blood pressure, *CAR* central activation ratio, *CF* central fatigue, *CO* cardiac output, *Gluc* glucose concentration, *HR* heart rate, *MVC* maximal voluntary contraction, *MVF* maximal voluntary force, *NA* not available, *PF* peripheral fatigue, *PPO* peak power output, *PRL* blood prolactin concentration, *PV* plasma volume, *RH* relative humidity, *RPE* rating of perceived exertion, *RR* ratings of recovery, *SkBF* skin blood flow, *SR* sweat rate, *SV* stroke volume, *T*_*core*_ core temperature, *TD* thermal discomfort, *T*_*face*_ face skin temperature, *T*_*head*_ head skin temperature, *T*_*local*_ local skin temperature, *T*_*neck*_ neck skin temperature, *TS* thermal sensation, *Tsk* skin temperature, *TT* time trial, *TTE* time to exhaustion, *VO*_*2*_ oxygen uptake, *VO*_*2max*_ maximal oxygen uptake, *Wc* water consumed

Although the combined cooling intervention may not be appropriate as a per-cooling strategy for both endurance and team sport athletes in the heat, it may be effective as a post-cooling strategy after performing exercise in the heat because it may compensate for the withdrawal of the thermoeffector, thereby reducing the magnitude of post-exercise hyperthermia. In particular, this intensive cooling intervention may benefit occupational workers wearing protective clothing or American football players wearing multiple layers of body armor more than neck cooling alone. However, this separate and combined effect has not been investigated and thus warrants further investigation.

## Practical Considerations

Summarizing the current literature to date (Tables 1, 2, 3, 4), neck cooling is preferred over head or face cooling because it can be used before and during exercise in the heat for both endurance and team sport athletes [66]. In addition, head cooling is preferred over facial cooling because it can also be used as a pre- and per-cooling strategy in the heat. It is worth mentioning that the cooling temperature selected from the neck cooling collar or cooling helmet must be individualized because each individual will have their own preferred cooling temperature, but the cooling temperature must be sufficient to alter the RPE without causing a further increase in core temperature. Furthermore, it is recommended that endurance athletes use either a neck or head cooling intervention following the completion of their race to compensate for the effect of post-exercise hyperthermia, especially when the heat stress environment is hot and humid [67].

Last, it is suggested that the cooling materials inside the cooling collar or the helmet should include phase change materials (including ice) rather than gel refrigerants because phase change materials provide a more stable cooling intensity during phase change than gel refrigerants [68]. Although soft gel could provide good flexibility when applied to the body surface, its temperature rises throughout the entire application period. Soft gel refrigerants, in particular, provide the most powerful cooling intensity at first, but this cooling power gradually diminishes over time. Furthermore, the mass of phase change materials should be large to provide athletes with prolonged cooling during exercise. Other cooling strategies such as the use of wearable cooling fans and a liquid cooling neck collar may be applicable; however, their actual effectiveness on performance improvement in exercising athletes requires further investigation.

## Directions for Future Research

While current studies have eloquently addressed the effects of neck, head, face as well as the combined head and neck cooling on both endurance and team sport performance, there are three major unexplored issues that warrant further investigation. First, recent studies have not specifically included evaluations of the effects of different cooling modes on local and whole-body thermal discomfort, thermal pleasantness and skin wettedness in their research design. It is well known that thermal perceptions can influence RPE, thereby affecting our physical performance in the heat [12, 69]. Furthermore, because those perceptual variables are important in determining our thermoregulatory behavior [12, 70, 71], it would be novel to investigate whether cooling per se would mask the signal to initiate our thermoregulatory behavior, which has an unlimited capacity to regulate our body temperature.

Second, previous studies have not taken into account the role of aerobic fitness in the effect of neck, head, face and combined head and neck cooling during self-paced exercise in heat. When exercising in the heat, well-trained populations are more accustomed to thermal discomfort [72] than untrained individuals, and thus, additional cooling may not be ergogenic when compared to untrained individuals. Indeed, previous studies primarily targeted endurance or team sport athletes, which could explain why some studies found no performance benefit from using neck cooling during exercise.

Next, the rationale for selecting cooling devices in a specific sports and exercise setting has not yet been thoroughly investigated. For instance, the temperature of the cooling device varies considerably, ranging from 21 to − 80 °C, and such variations in cooling temperature would undoubtedly alter our thermal perceptions and thus our exercise performance in the heat. Furthermore, to date, the selection of cooling temperatures is not evidence-based; rather, they are chosen without any supporting evidence for either thermoregulatory or perceptual response to heat. Additionally, the mass of cooling materials, the type of cooling strategy (e.g., air cooling, phase change material cooling, evaporative cooling, and liquid cooling) and the design of the cooling devices (e.g., packing material, insulation between the cooling material and human skin, and contact area) are unclear. It is also well known that exposure to high levels of solar radiation reduces endurance capacity and raises skin temperature during heat-related exercise. Nonetheless, previous cooling studies did not take into account the effects of sunlight on cooling during exercise-heat stress. Furthermore, under such conditions, the cooling materials (phase change materials and gel refrigerant) melt faster than in the absence of sun exposure. The aforementioned factors may have a significant impact on the cooling intensity and the cooling duration of the chosen device. As a result, their actual impact on performance enhancement while exercising in different heat stress environments may vary greatly. Therefore, the aforementioned issues merit further investigation because they would broaden our understanding of the effects of per-cooling in both compensable and uncompensable heat stress environments. Furthermore, only 12 out of 49 studies (see Tables 1, 2, 3, 4) included females as exercising participants. Females have very different thermal and perceptual responses to exercise heat stress across their menstrual cycle [73]. As a result, more research is needed in this area to investigate sex differences in thermal responses to head, face and neck cooling.

Last﻿, in addition to the head, neck and face cooling discussed in this narrative review, other per-cooling (cooling during exercise) strategies, such as palm cooling, have also been used to mitigate heat strain in athletes during heat. Human hands, in general, have the second highest cold thermosensitivity after the human facial regions (including the neck) [74]. The proximal regions of the hands, such as the palm and sole, are much more thermally sensitive than the rest of the hand regions [75]. Currently, only four studies on the use of palm cooling to improve exercise performance in heat have been found. According to Grahn et al., cooling the palm increased the endurance exercise duration by 42.7% and attenuated the temperature rise in the esophagus [76]. The effect of cooling on exercise endurance decreased as the workload increased. Grahn et al. [77] discovered that palm cooling could improve high-intensity resistive exercise, but the cooling benefits are dependent on many factors, including fitness levels, inherent athletic ability, and the types and durations of the training regimes. Palm cooling is recommended as a useful strategy for athletes to maximize the training effects of resistive exercise. Despite the abovementioned ergogenic benefits of palm cooling for subjects, two studies [78, 79] found conflicting results after cooling subjects at the palm in the heat. Amorim et al. [78] and Scheadler et al. [79] observed that palm cooling does not reduce thermal strain or improve endurance performance during exercise. Several marathon runners and race walkers ran with ice packs in their hands during the Tokyo 2020 Olympic Games and the Doha 2019 IAAF (International Association of Athletics Federations) World Athletics Championships. It is unclear whether palm cooling, such as by hand-holding ice packs, can improve endurance performance because existing studies did not use the time trial approach or the time to exhaustion in well-trained athletes. This possibility merits further investigation. In addition, no research has been conducted to investigate the effect of palm cooling on athletes’ perceptual responses. More research is needed to investigate this effect.

## Conclusions

Neck, face and head cooling could significantly reduce local skin temperature, improving local perceptual sensations such as thermal sensation and thermal discomfort in athletes exercising in the heat. For both endurance and team sport athletes, neck cooling is a better per-cooling strategy than head or face cooling. Furthermore, while head cooling is preferred over face cooling for endurance athletes, its effectiveness among team sport athletes has yet to be determined. For all athletes who have access to water, face cooling is a recommended cooling strategy. Furthermore, it is unclear whether the combined cooling of the head, neck and/or face could have a synergistic effect on sports and exercise performance in the heat. Future research should thoroughly investigate the potential of neck, head and face cooling to improve the performance of male and female athletes, while they participate in various types of sports and exercise activities in heat. Furthermore, the development of powerful but portable head, neck and face cooling systems is critical due to the great need to dissipate metabolic heat production during intense sports activities.

## Data Availability

Data are available on request from the authors.

## Abbreviations

Bla: Blood lactate concentration
BP: Blood pressure
CAR: Central activation ratio
CF: Central fatigue
CO: Cardiac output
FVR: Forearm vascular resistance
Gluc: Glucose concentration
HEX: High-intensity exercise
HR: Heart rate
IAAF: International Association of Athletics Federations
iEMG: Integrated electromyography
MVC: Maximal voluntary contraction
MVF: Maximal voluntary force
NA: Not available
PF: Peripheral fatigue
PPO: Peak power output
PRL: Blood prolactin concentration
PSI: Physiological strain index
PV: Plasma volume
RER: Respiratory exchange ratio
RH: Relative humidity
RPE: Rating of perceived exertion
RR: Ratings of recovery
SkBF: Skin blood flow
SR: Sweat rate
SV: Stroke volume
*T*_core_: Core temperature
TD: Thermal discomfort
*T*_face_: Face skin temperature
*T*_forehead_: Forehead skin temperature
*T*_head_: Head skin temperature
*T*_local_: Local skin temperature
*T*_neck_: Neck skin temperature
TPS: Total performance score
TS: Thermal sensation
Tsk: Skin temperature
*T*_ty_: Tympanic membrane temperature
TT: Time trial
TTE: Time to exhaustion
VE: Minute ventilation
VO_2_: Oxygen uptake
VO_2max_: Maximal oxygen uptake
Wc: Water consumed
WL: Weight loss
YYIR1: Yo–Yo intermittent recovery level 1 test
